# Interaction Mechanism and Loss Analysis of Mixing between Film Cooling Jet and Passage Vortex

**DOI:** 10.3390/e24010015

**Published:** 2021-12-22

**Authors:** Ziyu Chen, Kexin Hu, Yinbo Mao, Xinrong Su, Xin Yuan

**Affiliations:** Key Laboratory for Thermal Science and Power Engineering of Ministry of Education, Department of Energy and Power Engineering, Tsinghua University, Beijing 100084, China; chenziyu18@mails.tsinghua.edu.cn (Z.C.); hkx21@mails.tsinghua.edu.cn (K.H.); yimao@syr.edu (Y.M.); yuanxin@mail.tsinghua.edu.cn (X.Y.)

**Keywords:** gas turbine, film cooling, passage vortex, entropy generation rate

## Abstract

The interaction between the film-cooling jet and vortex structures in the turbine passage plays an important role in the endwall cooling design. In this study, a simplified topology of a blunt body with a half-cylinder is introduced to simulate the formation of the leading-edge horseshoe vortex, where similarity compared with that in the turbine cascade is satisfied. The shaped cooling hole is located in the passage. With this specially designed model, the interaction mechanism between the cooling jet and the passage vortex can therefore be separated from the crossflow and the pressure gradient, which also affect the cooling jet. The loss-analysis method based on the entropy generation rate is introduced, which locates where losses of the cooling capacity occur and reveals the underlying mechanism during the mixing process. Results show that the cooling performance is sensitive to the hole location. The injection/passage vortex interaction can help enhance the coolant lateral coverage, thus improving the cooling performance when the hole is located at the downwash region. The coolant is able to conserve its structure in that, during the interaction process, the kidney vortex with the positive rotating direction can survive with the negative-rotating passage vortex, and the mixture is suppressed. However, the larger-scale passage vortex eats the negative leg of the kidney vortices when the cooling hole is at the upwash region. As a result, the coolant is fully entrained into the main flow. Changes in the blowing ratio alter the overall cooling effectiveness but have a negligible effect on the interaction mechanism. The optimum blowing ratio increases when the hole is located at the downwash region.

## 1. Introduction

With the growth of the thermal efficiency of the gas turbine and the aero-engine, turbine inlet temperature increases, exceeding the metal melting point. Therefore, for consideration of the operation reliability, film cooling is widely adopted in modern cooling design to lower the surface temperature of the turbine component. There have been many factors validated to affect the film cooling effectiveness [1,2]. They are mainly classified by Bogard and Thole [3] into three aspects, including the coolant/mainstream conditions [4], the hole geometry [5,6] and the turbine geometry [7]. Among the existing studies, turbine endwall film cooling is one of the most challenging topics due to the complicated interaction between the cooling jet and the secondary flow structures in the endwall region.

To help understand the secondary flows, researchers have carried out many flow visualization experiments and some representative endwall flow topologies have been proposed by Hawthorne et al. [8], Langston [9], Sieverding et al. [10], Sharma et al. [11], Wang et al. [12] and Papa [13]. As reviewed by Sieverding [14], secondary flows in turbine passages can be summarized into two flow structures of the vortex structures and the endwall boundary layer. Their intensity is significantly influenced by the characteristics of the passage inlet boundary layer. The oncoming boundary layer rolls up in front of the blade leading edge and forms into a vortical motion, called the horseshoe vortex. The vortex structure separates into two legs, labeled the “pressure” leg and the “suction” leg. The pressure leg migrates from the pressure side to the suction side. During the migration, the pressure leg is fed by the endwall flow and then becomes the passage vortex. The suction leg stays close to the suction surface. Its rotation sense is opposite to that of the passage vortex.

At the same time that turbine endwall flow field attracted attention, the endwall film cooling obtained popularity. Blair [15] is commonly conceived to be the first who conducted experiments on the endwall film cooling. They validated the effect of the secondary flows on the endwall heat transfer and the film cooling effectiveness. Wright et al. [16] experimentally analyzed cooling effectiveness distributions on the blade platform with discrete film hole flows. They observed that due to the entrainment of the passage vortex, traces of coolant are weakened. Similar results are represented by Shiau et al. [17] that a low-effectiveness curve band is observed close to the vane suction side, and it can be identified as the lift-off line of the passage vortex. Friedrichs et al. [18,19,20] systematically studied the interaction mechanism between the secondary flows and the cooling jet. They found that trajectories of the coolant are sensitive to changes of the outlet flow field by measuring distributions of the local film cooling effectiveness using the ammonia and diazo technique. Under the effect of the endwall crossflow, coolant turns the direction from the pressure side towards the suction side regardless of the hole exit angle. For holes beneath the lift-off line, the coolant is entrained into the passage vortex before it can provide cooling protection onto the endwall surface. Reversely, Friedrichs observed the phenomenon that the high-momentum injection upstream of the lift-off line is able to suppress the development of the crossflow, which can help improve the aerodynamic efficiency. Thomas et al. [21] further pointed out a critical momentum ratio *I* = 1.71 that intensity of the secondary flow can be restrained when *I* is larger than 1.71. They revealed that the phenomenon of the secondary flow can be negligible when *I* > 6.4.

The existing researches paid attention to the overall cooling performance of the full-coverage film cooling onto the turbine endwall. However, many factors contribute to the complexity of the endwall film cooling, including the passage vortex, the crossflow, the pressure gradient, the hole-to-hole interaction, and so on. There lack effective ways to reveal their influences separately. Li et al. [22] introduced a curved channel to simulate the formation of the endwall crossflow. By this means, the influence of the crossflow on the film cooling performance can be quantified. They compared the effectiveness distributions with those of the straight channel (without crossflow) and concluded that the crossflow can redirect the cooling jet and enhance lateral spreading. This experimental facility is similarly adopted by Miao et al. [23] to analyze the crossflow effect on the purge air cooling. However, there lacks sufficient attention to the interaction between the vortex structure and cooling injections. Ligrani et al. [24,25,26] firstly located a vortex generator upstream of a row of cylindrical film cooling holes to illustrate the effects of vortices on heat transfer and injectant distributions. They divided the vortex-affected region into the downwash region and the upwash region by observing the directions of the velocity vectors. The downwash flow thins the boundary layer beneath and accumulates coolant near the upwash side where augmented film cooling protection can be obtained. Similar researches are carried out by Jung et al. [27] and Zhao et al. [28]. They qualitatively compared cooing effectiveness distributions to investigate the effects of the relative location of the vortex to the cooling hole. When the vortex core passes over the center of the film hole, the injectant can be swept away, resulting in a triangular low-effectiveness region. When the downwash side is above the hole exit, coolant is pushed down to the wall, thus enlarging film coverage. There are some precedent relevant researches, but there still lack quantitative assessments on the effect of the passage vortex on the film cooling performance. The effect of the vortex on film cooling is attributed to the superposition of the first-order velocity field while the vorticity field is second-order. The vortical interaction between the passage vortex and injection kidney vortices should also be considered which is believed to play a significant role.

To sum up, the cooling jet in the endwall region is affected by many parameters, including the complex vortex structures, the pressure gradients in both the transverse and streamwise directions, also the interaction between neighboring cooling jets. In most existing studies about endwall cooling, their influencing parameters coexist and it is difficult to analyze the effect of the individual parameter. The primary purpose of the current work is to study the effect of the passage vortex on the film cooling performance. The factor of the passage vortex is separated by placing a half-cylinder in a straight channel to generate the vortex structure, similar to its formation mechanism in the real engine but without the flow turning or acceleration. An analytic method based on the entropy generation rate is introduced to analyze the interaction process between the single hole film cooling jet and the passage vortex and reveal the underlying mechanism. It is found that if the cooling hole is suitably positioned, the cooling performance can be obviously improved, benefiting from the favorable interaction between the jet and the vortex.

The structure of the article is as follows. Section 2 introduces the numerical setup and the results are validated using the experimental data. Section 3 illustrates the flow field of the main-flow passage and the hole arrangement. The analysis method based on the entropy generation rate is introduced in Section 4. The interaction mechanism between the passage vortex and the film cooling is analyzed in Section 5. The last section is the conclusion.

## 2. Numerical Setup and Validation

**Figure 1:**
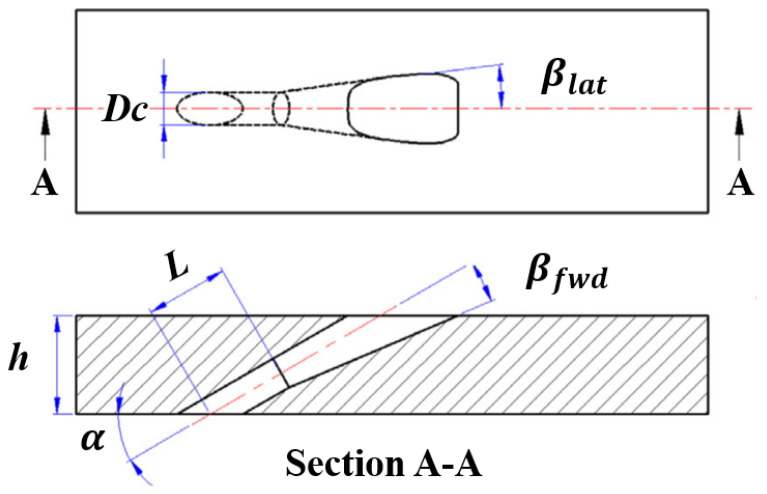
Geometry of 7-7-7 shaped hole.

In the current section, the numerical setup is introduced and the calculation results are validated using the experimental data in the open literature. The 7-7-7 shaped hole designed by Schroeder et al. [29] is selected to quantify changes in its cooling effectiveness with and without the effect of the vortex structure. The film cooling hole diameter is 7.75 mm. Detailed geometry of the 7-7-7 shaped hole is illustrated in Figure 1. The injection angle between the hole axis and the main-flow streamwise direction is $30^{\circ }$. The laidback angle and the two expansion angles are all $7^{\circ }$, that is why this hole shape is called the 7-7-7 shaped hole.

**Figure 2:**
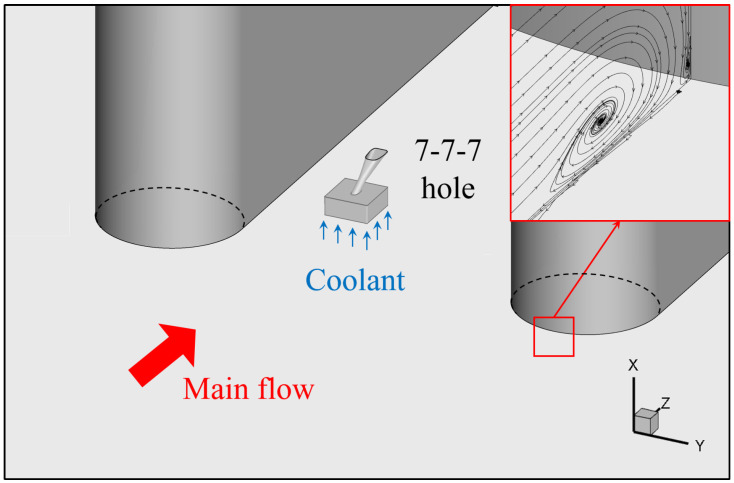
Illustration of the calculation model.

**Figure 3:**
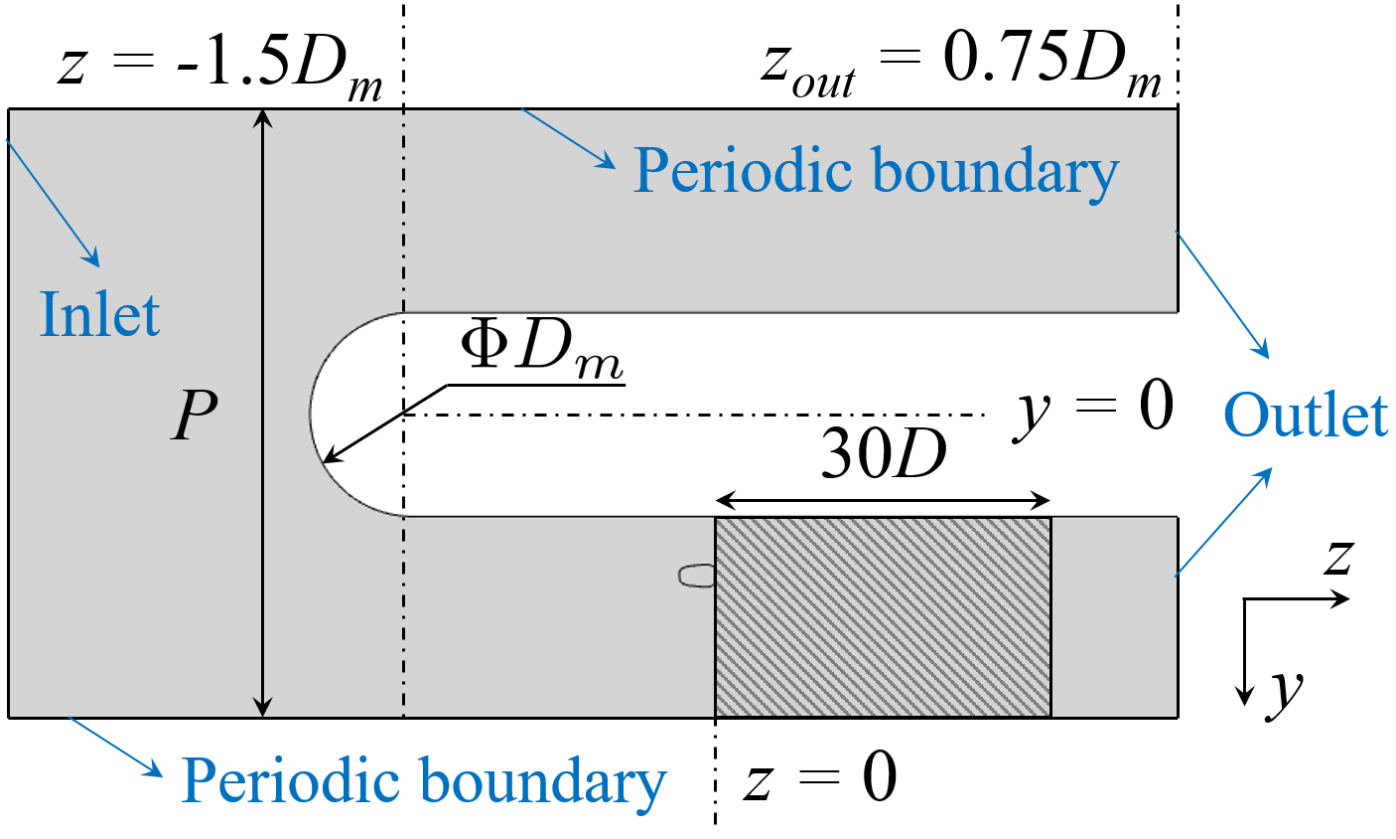
Top view (X direction) of the calculation domain.

In the current study, to analyze the effect of the vortex structure on film cooling performance, a half-cylinder is introduced to generate the horseshoe vortex, and the computational model is shown in Figure 2. One flow passage with periodic boundaries on both sides is included in the calculation domain to reduce the computation cost. The top view of the final calculation domain is illustrated in Figure 3. Axis X represents the spanwise direction while Y and Z, pitchwise and streamwise directions respectively. The cylinder diameter $D_{m}$ is 150 mm and the pitchwise distance *P* is 450 mm. The length ratio between the cylinder diameter and the hole diameter is close to 20, similar to that of the geometry in the turbine cooling design. The main-flow velocity $U_{0}$ is equal to 22 m/s at the inlet where the turbulence intensity is 5% and the turbulence length scale is estimated to be 10 mm. The calculated Reynolds number based on the leading edge diameter, $Re_{Dm}=2\times 10^{5}$, is within the range of the real engine conditions. The outlet boundary is open to the atmosphere. The calculation medium in the current study is the ideal gas with constant specific heat, thermal conductivity and viscosity. The inlet temperature of the main flow is set at 300 K and the coolant inlet temperature is 200 K to ensure the density ratio DR = 1.5. The coolant is supplied by a stagnation plenum, whose cross section is 36$D_{c}^{2}$. Six different blowing ratios *M* from 0.5 to 3.0 are included. The relevant parameters are also listed in Table 1 for convenience.

**Figure 4:**
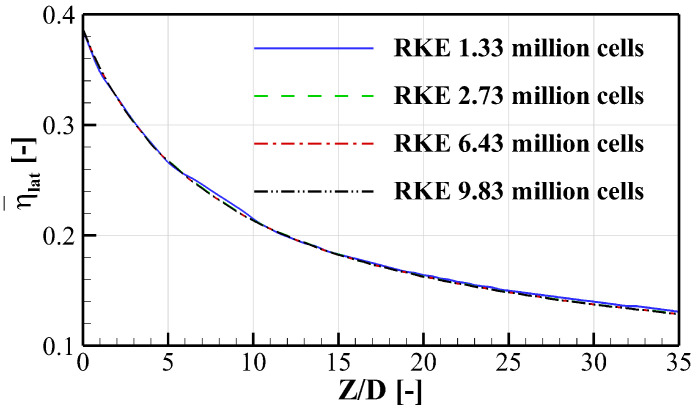
Grid independence analysis of laterally averaged adiabatic cooling effectiveness ${\bar{\eta }}_{lat}$.

The steady RANS (Reynolds Averaged Navier-Stokes) equations are employed in the current study. The numerical simulations are solved using ANSYS Fluent 19.2, where the “Coupled” pressure velocity coupling scheme and second-order discretization for all variables are adopted [30]. For the grid independence analysis, the film cooling over a flat plate is considered. To obtain the proper mesh density, four different mesh sizes of 1.33 million, 2.73 million, 6.43 million, and 9.83 million cells are selected to compare cooling effectiveness distributions. To be noted, the adiabatic cooling effectiveness η is defined in part of the NOMENCLATURE to quantify the cooling performance. Figure 4 plots the laterally averaged cooling effectiveness ${\bar{\eta }}_{lat}$ (over 6*D* in the lateral direction) distributions along the streamwise direction. It can be seen that there is no significant difference between meshes. The mesh size of 2.73 million cells is selected which is of sufficiently high quality to predict the cooling performance and its differences between larger-size meshes are negligible.

**Figure 5:**
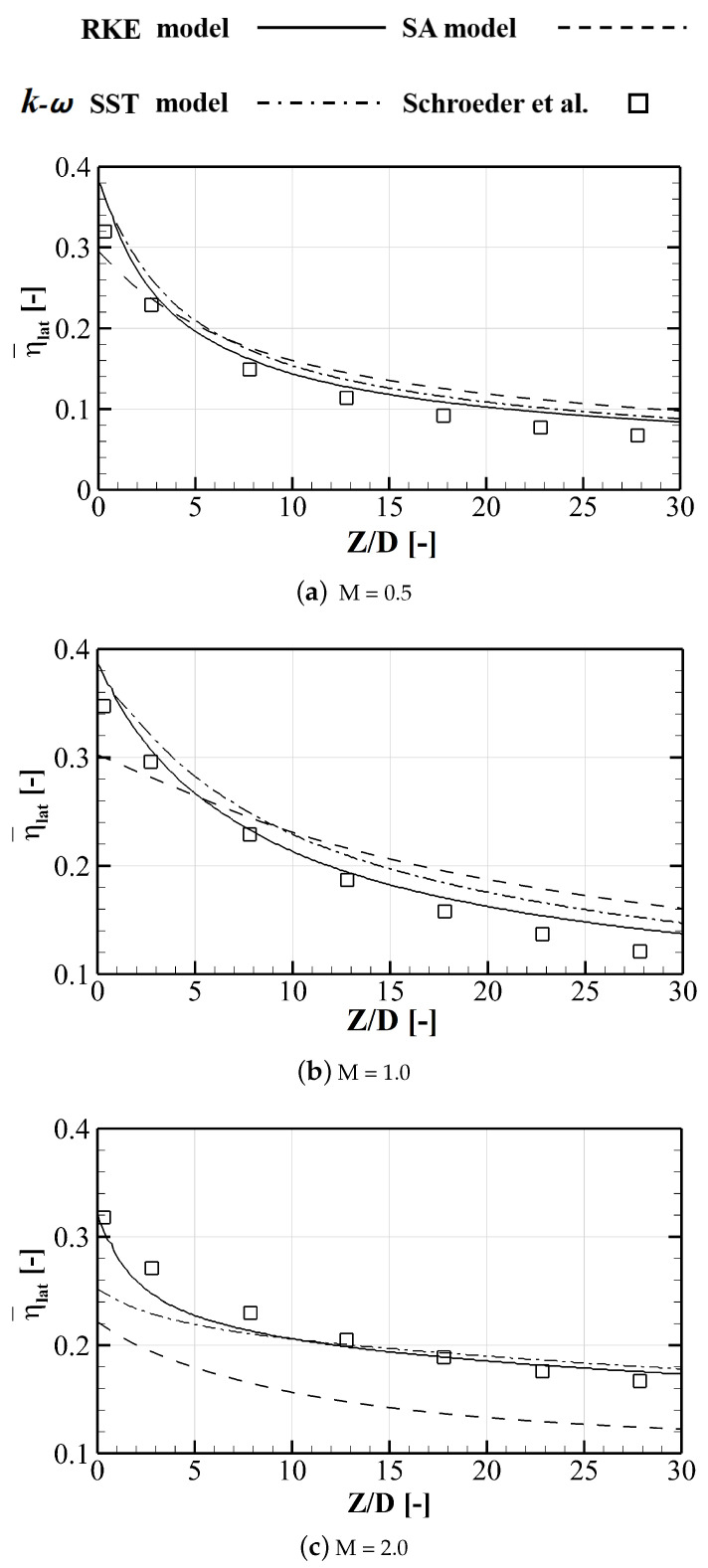
Validation of the laterally averaged film cooling effectiveness ${\bar{\eta }}_{lat}$ using the experimental data by Schroeder et al. [29].

**Figure 6:**
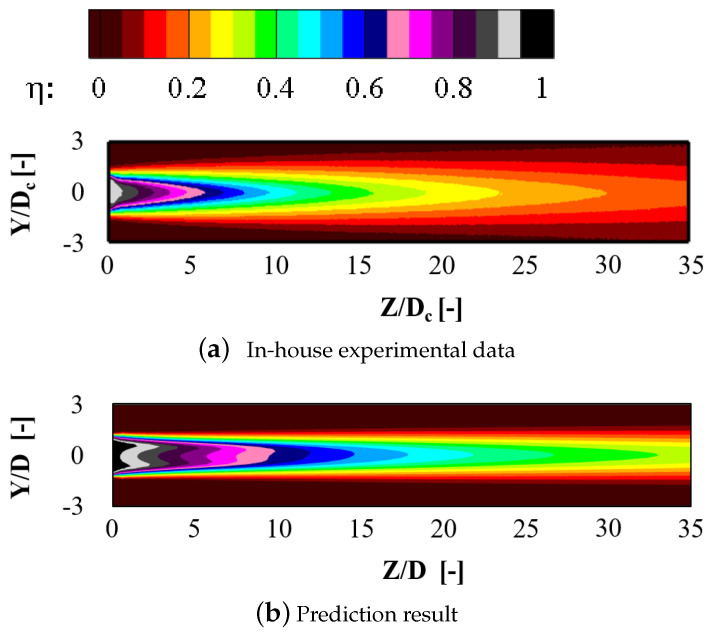
Comparisons of effectiveness distributions between numerical results and experimental data (*M* = 1.0).

Three different turbulence models including the Realizable k-ε (RKE), the Spalart-Allmaras (SA), and the *k*-ω SST model are examined to compare their results with the experimental data by Schroeder et al. [29]. The laterally averaged effectiveness distributions are plotted in Figure 5. The symbols denote the experimental data and different line patterns represent different turbulence models. There are three blowing ratios included here for the numerical validation. Results come that the RANS simulation with the RKE model can well predict the streamwise distributions of the cooling effectiveness. Based on the above numerical validation analysis, the RANS method with the RKE model is applied. Further comparisons of the two-dimensional effectiveness distributions between numerical results and experimental data are contoured in Figure 6. It can be observed that the numerical result underestimates the coolant lateral spreading by RANS. The phenomena are commonly observed in existing researches [31,32]. However, it is believed that the turbulence model would be very useful in revealing the effect of the passage vortex on the cooling performance.

**Figure 7:**
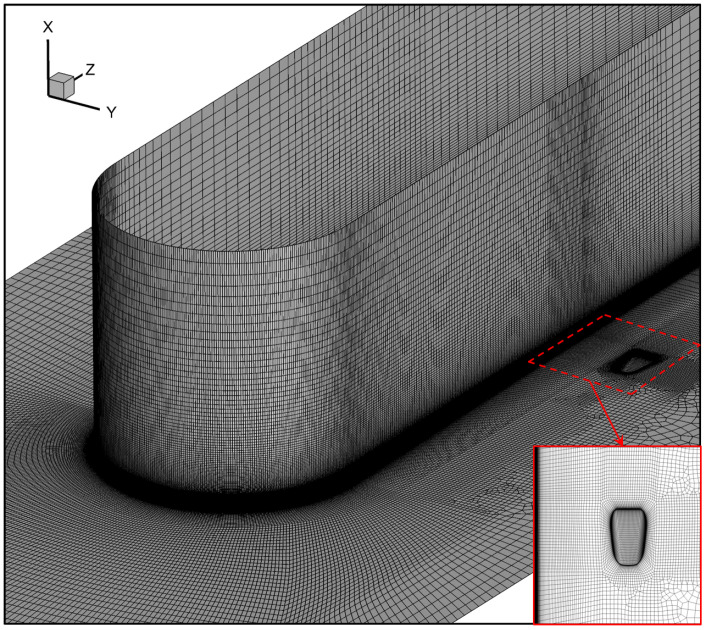
Final calculation mesh, including mesh details near the hole exit.

For the calculation domain in Figure 2, the final mesh size is 9.5 million cells including meshes for the near hole region and the main-flow passage, as illustrated in Figure 7. O-type meshes are generated at the hole exit and around the wall of the half-cylinder to improve the grid quality. To accurately capture the hole viscous sublayer, the dimensionless parameter Y+ at the first off-wall mesh layer is less than 1.0. The mesh independence analysis on the main flow is also conducted to endure that it is of sufficient quality to accurately calculate the formation and development of the passage vortex. The convergence criterion is that the temperatures of the local monitoring points do not change more than 0.01% for more than 500 iterations. The residuals for all parameters drop for more than five orders of magnitude and the residual for energy, eight orders when the simulation has converged.

**Figure 8:**
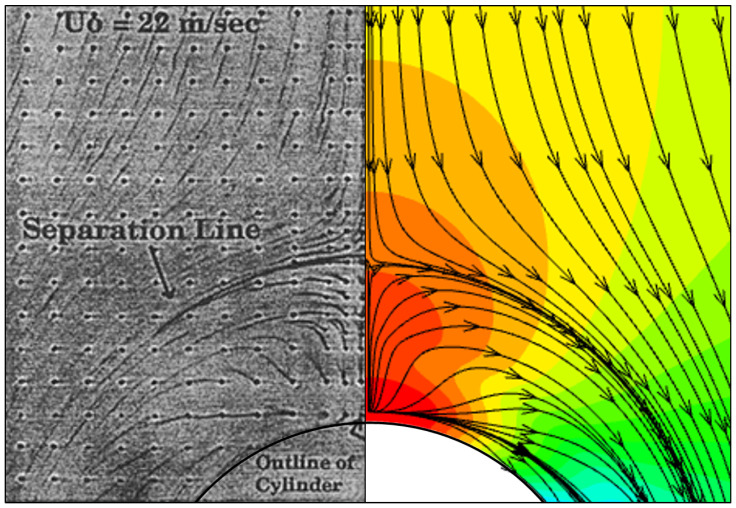
Illustration of the endwall limiting streamlines (left: experimental result [33]; right: CFD result in current study).

This numerical method is further validated using the endwall limiting streamlines to ensure that it suits the aerodynamic performance predictions. Results are shown in Figure 8. The CFD result lies on the right side. The flow visualization result on the left is obtained by Eckerle et al. [33] experimentally. In the current study, the simulation setup is consistent with that of the experiment for comparison. The limiting streamlines indicate the directions of the near-wall flow velocity. For the leading edge region of the cylinder, they represent the spatial scale of the horse-shoe vortex during its formation. By qualitative comparison, the RANS with the RKE model can well predict the generation of the vortex structure.

## 3. Flow Field in the Main-Flow Passage

The current section briefly illustrates the flow field in the main-flow passage and the hole arrangements for further analyses.

**Figure 9:**
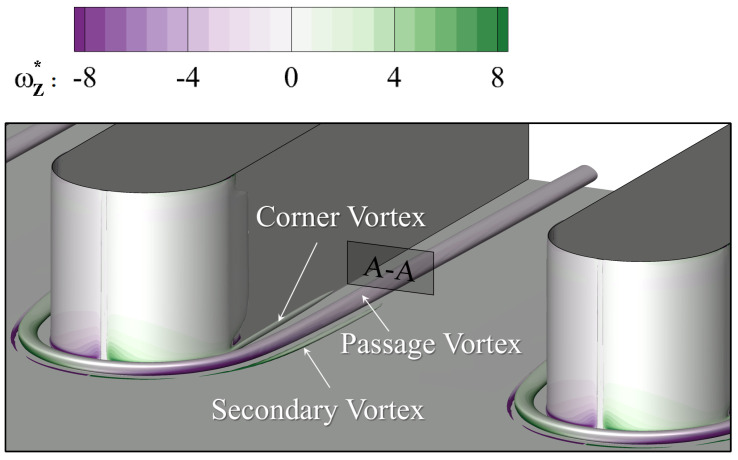
Iso-surface of the Q-criterion in the main-flow passage.

**Figure 10:**
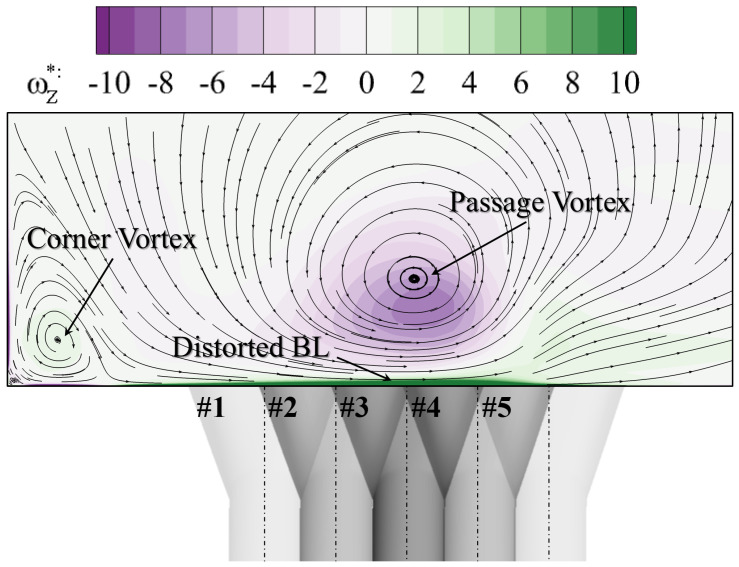
Vortex structures at A-A slice in Figure 9.

Figure 9 visualizes the vortex structures using the iso-surface of the Q-criterion. It is contoured using the non-dimensionalized Z-direction vorticity to show its sense of rotation. The horseshoe vortex forms in front of the leading edge and migrates following the direction of the main flow. To be clarified that this vortex in the passage is then labeled “passage vortex” (PV) in the current work, in that the main object of the study is to reveal the effect of the passage vortex on film cooling in the endwall region. The corner vortex and the secondary vortex can also be observed. However, their intensity is weaker and dissipates along the streamwise direction. The slice A-A is located at the trailing edge of the cooling hole. Figure 10 contours the vorticity distribution at the slice A-A. The vector lines identify directions of the local velocity, similar to streamlines. The corner vortex stays close to the blade surface and a much smaller vortex structure is induced in the corner region. Due to their weak vorticity intensity, they will not be considered in the current study. The large-scale passage vortex dominates the flow field in the passage. It distorts the beneath boundary layer and induces vorticity with opposite rotation direction. The boundary layer is labeled “BL” in Figure 10. These characteristics will directly influence the flow field at the outlet of the cooling hole.

As has been revealed in Section 1, the flow field in the turbine endwall region is rather complex. Apart from the passage vortex, the streamwise flow acceleration and the crossflow derived from lateral pressure gradient also have significant effects on the film cooling performance [22,34]. In the current study, the intensity of the streamwise acceleration and the crossflow is quantified to demonstrate that the effect of the passage vortex can be specially separated by placing the half-cylinder onto a straight channel.

The acceleration parameter *K* is introduced to quantify the intensity of the flow acceleration [34]:

(1) $$K=\frac{\nu_{0}}{U_{0}}\frac{\partial U_{z}}{\partial z}$$

**Figure 11:**
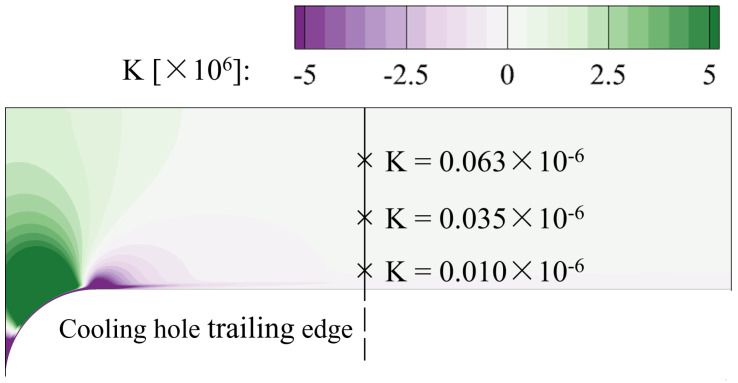
Distribution of the acceleration parameter at the mid-span slice.

$\nu_{0}$ and $U_{0}$ are the main-flow viscosity and velocity. The z-coordinate denotes the streamwise direction. The distribution of *K* at the mid-span slice is contoured in Figure 11. The black line marks the streamwise location of the cooling hole trailing edge. The *K* value is less than $0.1\times 10^{-6}$ for the region near and downstream the cooling hole. The effect of the streamwise pressure gradient can be eliminated in the current study.

In the turbine passage, the flow turning results in the lateral pressure gradient from the pressure side towards the suction side. In the near-wall region, the centrifugal force fails to balance this pressure difference, generating the crossflow, which also heavily affects the cooling jet [22]. In the current study, the straight passage with a constant width is analyzed where negligible flow turning is observed. The deviation angle γ is defined as:

(2) $$\gamma =tan^{-1}\left(\frac{U_{y}}{Uz}\right)$$

**Figure 12:**
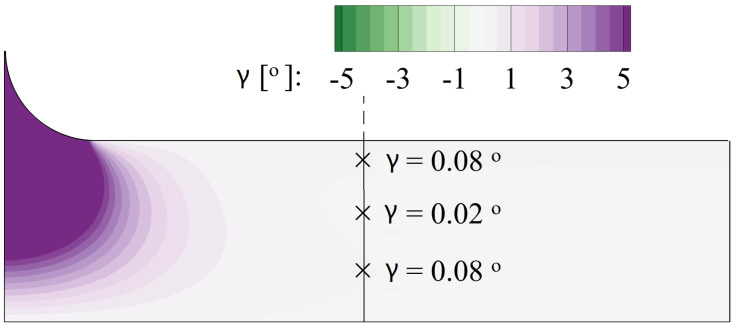
Distribution of the deviation angle at the mid-span slice.

The distribution at the mid-span slice is contoured in Figure 12. It can be validated that the calculation domain is free from the effect of the crossflow.

In the current work, a single hole is analyzed to reveal the mechanism of coolant migration under the effect of the passage vortex, excluding the hole-to-hole interaction. According to the above results about *K* and γ, the effects of streamwise and transverse pressure gradients are also minimized. As a result, with the current simplified geometry, the interaction mechanism between the jet and the vortex can be separated from the other parameters.

The streamwise position of the cooling hole can be seen in Figure 3. For the pitchwise direction, five relative positions are included labeled from “#1” to “#5” on the upper left in Figure 10 at intervals of 1.0D. The #3 hole locates beneath the core of the passage vortex. The first two locations are in the downwash region of the vortex and the last two in the upwash region, as defined by Ligrani et al. [24,25,26]. The effect of the hole position will be discussed in the following section.

## 4. Entropy Generation Rate Based Loss Analysis Method

In the previous analyses on the injection/main flow analyses, the global variables, for example of the pressure loss coefficient [19,35], the entropy production [36] and the adiabatic cooling effectiveness [37], are selected to quantify the aerodynamic efficiency and the cooling performance. However, they reflect the historic effect inside the passage and, thus lack the ability to provide more information about where the loss occurs [38] in the turbine passage. In the current study, the analysis method based on the entropy generation rate ($\dot{S}$) is introduced to effectively quantify the intensity of local irreversible processes. This method is applied to analyses on the reference case of the flat plate film cooling and its advantage in revealing the dynamic mechanism is validated. Based on the scale analysis on the magnitude, the irreversible heat transfer dominates the injection/mainstream interaction process in the current study.

### 4.1. Entropy Generation Rate

The entropy generation rate quantifies the intensity of the irreversibility loss. Based on the second law of thermodynamics, there are two sources of irreversibility in the flow field, which are caused by the irreversible heat transfer and by the fluid viscosity [39].

Driven by the local temperature gradient, the entropy generation during the irreversible heat transfer process is labelled as “${\dot{S}}_{ther}$”. For the incompressible fluid without internal heat generation, the volumetric rate of entropy generation [40] can be expressed as:

(3) $${\dot{S}}_{ther}=\frac{k}{T^{2}}\;\cdot \;\frac{\partial T}{\partial x_{j}}\frac{\partial T}{\partial x_{j}}$$

where the thermal diffusion conductivity *k* is 0.0242 W/(m·K). Considering the effect of the turbulent diffusion, the Equation (3) can be written as:

(4) $${\dot{S}}_{ther}=\frac{k+k_{t}}{T^{2}}\;\cdot \;\frac{\partial T}{\partial x_{j}}\frac{\partial T}{\partial x_{j}}$$

where the turbulent thermal diffusion conductivity $k_{t}$ can be calculated by:

(5) $$k_{t}=\rho \;\cdot \;C_{p}\;\cdot \;\alpha_{t}$$

The turbulent thermal diffusion coefficient $\alpha_{t}$ is

(6) $$\alpha_{t}=\frac{\nu_{t}}{Pr_{t}}$$

where $\nu_{t}$ is the turbulent viscous diffusion coefficient and the turbulent Prandtl number $Pr_{t}$ = 0.85 is suggested in the RANS simulation. Then, the volumetric rate of thermal entropy generation can be calculated, and the non-dimensionalized parameter $EGR_{ther}$ is defined as:

(7) $$EGR_{ther}=\left.{\dot{S}}_{ther}/\left(\frac{\rho_{0}\;\cdot \;s_{0}}{\tau_{c}}\right)\right.=\left.{\dot{S}}_{ther}/\left(\frac{\rho_{0}\;\cdot \;s_{0}\;\cdot \;U_{0}}{D}\right)\right.$$

Parameters with the subscript “0” denote variables of the inlet main flow. After the coolant is ejected from the cooling hole, heat is irreversibly transferred from the high-temperature main flow to the low-temperature coolant during the mixing process. Then, the coolant loses its cooling potential and the adiabatic cooling effectiveness is lowered along the streamwise direction.

The other source of the entropy generation is caused by the fluid viscosity. The equation can be similarly written as:

(8) $${\dot{S}}_{visc}=\frac{1}{T}\tau_{i,j}\varepsilon_{i,j}$$

$\tau_{i,j}$ denotes the viscous stress, considering both the laminar stress and the turbulent stress. $\varepsilon_{i,j}$ is the tensor of the deformation rate. This term is labeled as “$EGR_{visc}$” and it is non-dimensionalized in the same way:

(9) $$EGR_{visc}=\left.{\dot{S}}_{visc}/\left(\frac{\rho_{0}\;\cdot \;s_{0}}{\tau_{c}}\right)\right.=\left.{\dot{S}}_{visc}/\left(\frac{\rho_{0}\;\cdot \;s_{0}\;\cdot \;U_{0}}{D}\right)\right.$$

In the following sections, local distributions of the entropy generation rate accompanied by details of the flow structures can help reveal the underlying mechanism during the injection/mainstream interaction process.

### 4.2. Reference Flat Plate Film Cooling without Passage Vortex

In the current study, the flat plate film cooling at M=1.0 is also studied, to demonstrate the advantages of the entropy analysis method, also to provide comparative information for following analyses on the film cooling performance under the effect of the passage vortex.

**Figure 13:**
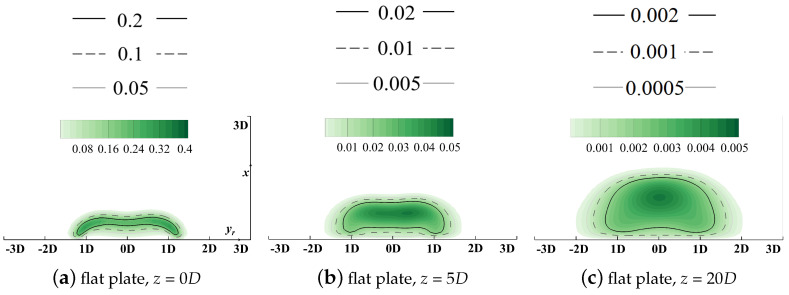
Contour of thermal entropy generation rate $EGR_{ther}$, flat plate film cooling at *M* = 1.0.

**Figure 14:**
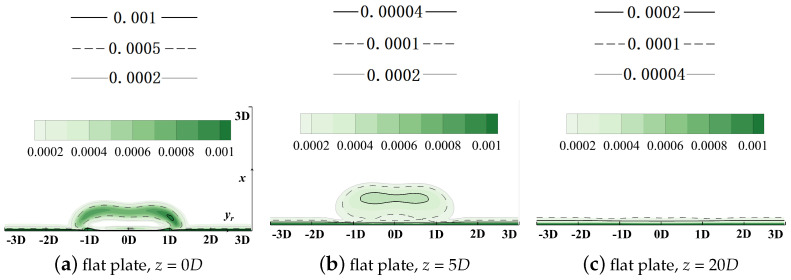
Contour of viscous entropy generation rate $EGR_{visc}$, flat plate film cooling at *M* = 1.0.

Streamwise slices at *z* = 0*D*, 5*D*, and 20*D* are selected to demonstrate local distributions of the volumetric entropy generation rate. Results are contoured in Figure 13 and Figure 14.

**Figure 15:**
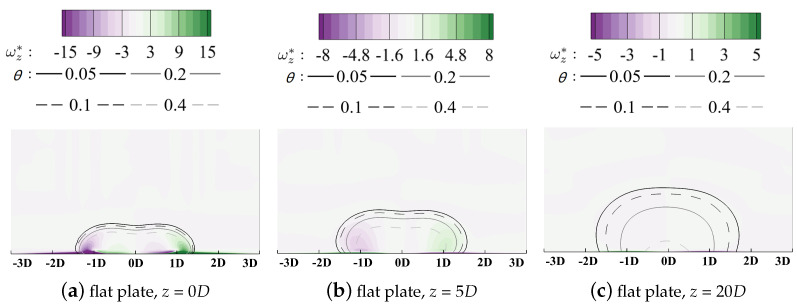
Contour of vorticity with isolines of non-dimensionalized temperatures, flat plate film cooling at *M* = 1.0.

The horizontal relative axis $y_{r}$ lies in the pitchwise direction and the vertical axis *x* is in the spanwise direction. It should be clarified that the original point of the $y_{r}$ axis denotes the pitchwise location of the cooling hole centerline, but its corresponding *y* value varies for different hole locations in the following sections. Distributions of the vorticity with isolines denoting values of the non-dimensionalized temperatures are contoured in Figure 15 to add details of the flow field and reveal the dynamic mechanism during the injection/mainstream interaction.

As illustrated in Figure 13, the thermal irreversible entropy generation occurs at the interface between the coolant and the outside main flow where a large temperature gradient is observed. Two high-heat-transfer regions are observed at $y_{r}$ = ±1D. These can be attributed to the existence of the kidney vortices (KV) in Figure 15a. KV entrains the high-temperature mainstream inside, thus enhancing the mixture process. The vortical structure dissipates significantly along the streamwise direction, and its effect is weakened. When the coolant fully attaches onto the wall surface, the heat transfer core region lies near the centerline where the vertical temperature gradient in *x* direction dominates the heat process compared with that in *y* and *z* direction. It should be clarified that due to the adiabatic thermal boundary conditions at the wall boundaries, the entropy generation due to the irreversible heat transfer in the near-wall region is suppressed.

Similar phenomena can be observed in Figure 14. After the coolant is ejected from the hole, severe shear actions occur at the main flow/coolant interface, and the velocity difference dissipates significantly. Then, the injection turns towards the direction of the main flow. However, it is noted that, apart from the main flow/cooling injection mixture, the viscosity effect inside the boundary layer also results in significant entropy generation and it dominates the flow filed downstream at the location z/D = 20, where negligible effect by the film cooling injection can be observed.

The above analyses validate the advantages of the entropy generation rate-based method in revealing the loss mechanism during the mixing process between coolant and the main flow. The counter-rotating vortex pair is emphasized to have a significant effect on the thermal and aerodynamic performance.

### 4.3. Scale Analysis on the Magnitude of Thermal and Viscous Entropy Generation Rates

It is noticed that magnitude of $EGR_{visc}$ is much smaller than $EGR_{visc}$ by comparing legends in Figure 13 and Figure 14. In the current section, the scale analysis on the magnitude of the thermal and viscous entropy generation rates is conducted to demonstrate which contributes more to the entropy generation during the injection/mainstream interaction process.

The thermal entropy generation rate can be calculated by Equation (4). Compared with the turbulent thermal diffusion conductivity, the effect of the laminar diffusion can be neglected. The temperature gradient can be estimated by the temperature difference (ΔT) divided by the characteristic length (*D*). The magnitude of the thermal entropy generation rate can be estimated by:

(10) $${\dot{S}}_{ther}∼\frac{k_{t}}{T^{2}}{\left(\frac{\Delta T}{D}\right)}^{2}$$

Similarly, the entropy generation rate by the fluid viscosity can be calculated by Equation (8). The velocity ratio in the turbine film cooling is on the magnitude of 1.0, and the velocity difference can be estimated by the main-flow velocity ($U_{0}$). Neglecting the laminar viscosity and the result can be written as:

(11) $${\dot{S}}_{visc}∼\frac{\mu_{t}}{T}{\left(\frac{\Delta U}{D}\right)}^{2}∼\frac{\mu_{t}}{T}{\left(\frac{U_{0}}{D}\right)}^{2}$$

Then, dividing the thermal entropy generation rate (Equation (10)) by the viscous thermal entropy generation rate, the scale analysis on the magnitude can be written as:

(12) $$\frac{{\dot{S}}_{ther}}{{\dot{S}}_{visc}}∼\frac{\frac{\Delta T}{T}\;\cdot \;\frac{\Delta T}{T}\;\cdot \;\frac{\rho C_{p}\alpha_{t}}{D^{2}}}{\frac{1}{T}\;\cdot \;\frac{\rho \nu_{t}U_{0}^{2}}{D^{2}}}$$

The relationship between $\alpha_{t}$ and $\nu_{t}$ can be referred to in Equation (6). The local temperature can be calculated using the local non-dimensionalized temperature θ:

(13) $$T=T_{0}-\theta \left(T_{0}-T_{c}\right)$$

$T_{0}$ and $T_{c}$ denote inlet temperatures of the main flow and the coolant. Then, by introducing the density ratio DR, Equation (12) can be written as:

(14) $$\frac{{\dot{S}}_{ther}}{{\dot{S}}_{visc}}∼\frac{1}{Pr_{t}}\frac{1}{\frac{1}{1-\frac{1}{DR}}-\theta }\frac{C_{p}\Delta T}{U_{0}^{2}}$$

DR in the real engine is generally on the order of 2.0, and equals to 1.5 in the current study. The value of the non-dimensionalized temperature θ is lower than 1.0. The turbulent Prandtl number is on the magnitude of 1.0. Equation (14) can be further simplified as:

(15) $$\frac{{\dot{S}}_{ther}}{{\dot{S}}_{visc}}∼\frac{\Delta T}{U_{0}^{2}/C_{p}}$$

The numerator is the temperature difference between the coolant and the main flow while the denominator denotes the kinetic energy. The temperature difference between the coolant and the main gas is set at 100 K, while the velocity difference is on the magnitude of 24 m/s in the current study. The final ratio calculated using Equation (15) is in the magnitude of $1\times 10^{2}$, similar to that in the real engine. The entropy generation rate due to the irreversible heat transfer is much larger than that by the fluid viscosity in the region dominated by the interaction between the jet and the main flow.

The proportion of the thermal entropy generation rate ${\dot{S}}_{ther}$ compared with the overall value ${\dot{S}}_{gen}$ is defined as the Bejan number Be [41]:

(16) $$Be=\frac{{\dot{S}}_{ther}}{{\dot{S}}_{gen}}=\frac{{\dot{S}}_{ther}}{{\dot{S}}_{ther}+{\dot{S}}_{visc}}$$

**Figure 16:**
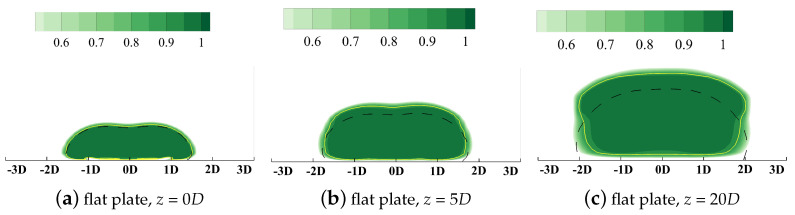
Distributions of the Bejan number Be denoting the proportion of the thermal entropy generation rate, flat plate film cooling at *M* = 1.0.

The distributions are contoured in Figure 16 which supports the scale analysis given above. The dashed line denotes the non-dimensionalized temperature value at θ=0.01, which can be deemed as the boundary of the cooling injection. The yellow solid line is the isoline where ${\dot{S}}_{ther}$ contributes 90% of the overall ${\dot{S}}_{gen}$. It can be observed that the thermal entropy generation dominates in the region where the cooling injection mixes with the main flow. Therefore, in the following sections, only the thermal irreversible entropy generation, ${\dot{S}}_{ther}$, will be included to analyze the loss of the cooling capacity during the mixing process. However, it should be clarified that, for the region inside the boundary layer and in the main-flow passage, the viscous effect still dominates. This coincides with the conclusions in the researches by Lin and Li [42,43] where no coolant is injected into the main flow.

### 4.4. Verification of the RANS-Solved Entropy Generation Rate

As concerned in some cases [42], RANS lacks the ability to accurately resolve small-scale flow details or transient structures, for example in the wake region. Then, some may worry about whether the calculation of EGR using RANS is reliable. Large eddy simulation (LES) and direct numerical simulation (DNS) are more accurate ways of resolving local entropy generation rates. However, in industrial problems, e.g., film cooling at varying blowing ratios and locations, the computation costs of LES and DNS are unacceptable. There have existed many attempts to adopt the entropy generation rate-based method by RANS [44,45,46,47]. As suggested by Jin et al. [39], the entropy generation rate is calculated using the turbulence production rate instead of the local dissipation rates, which is adopted in the current study. This ensures that the calculation of the entropy generation rate is independent of choices of turbulence models. Though there might exist some deviations derived from the defect of RANS, it provides a useful method in loss analyses for industrial applications.

**Figure 17:**
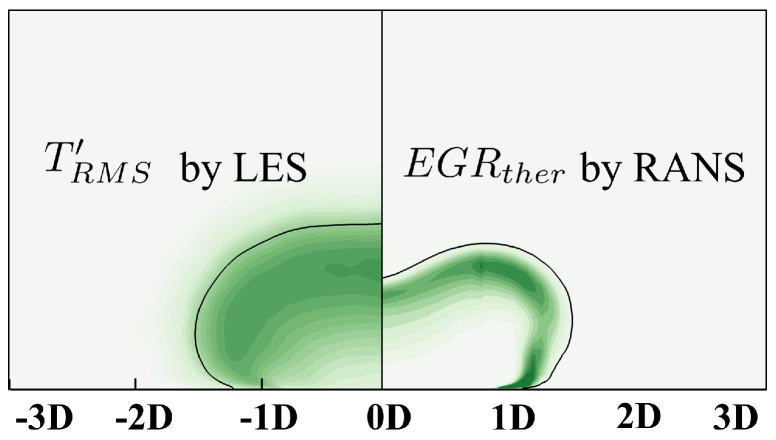
Demonstration of $T_{RMS}$ calculated by LES at *z* = 0*D*, *M* = 3.0 (the isoline of θ = 0.1).

Based on definition, EGR can be theoretically divided into two parts, contributed by the steady effect and the unsteady effect. The latter one is closely related to the turbulence fluctuation. Greater temperature fluctuation, for example, results in higher thermal irreversible mixture correspondingly. Therefore, EGR by RANS should be reasonably related to the fluctuation term, for example that resolved by LES. The results can reasonably stand on the conclusion that the RANS simulation is able to resolve the local high-EGR or low-EGR and reveal the underlying mechanism. Root-mean-square (RMS) fluctuating temperature and fluctuating velocity by high-accuracy LES are demonstrated to posteriorly support the entropy generation rate (EGR) calculation in the current study. The contour of the root mean square of the fluctuating temperature is illustrated in Figure 17.

**Figure 18:**
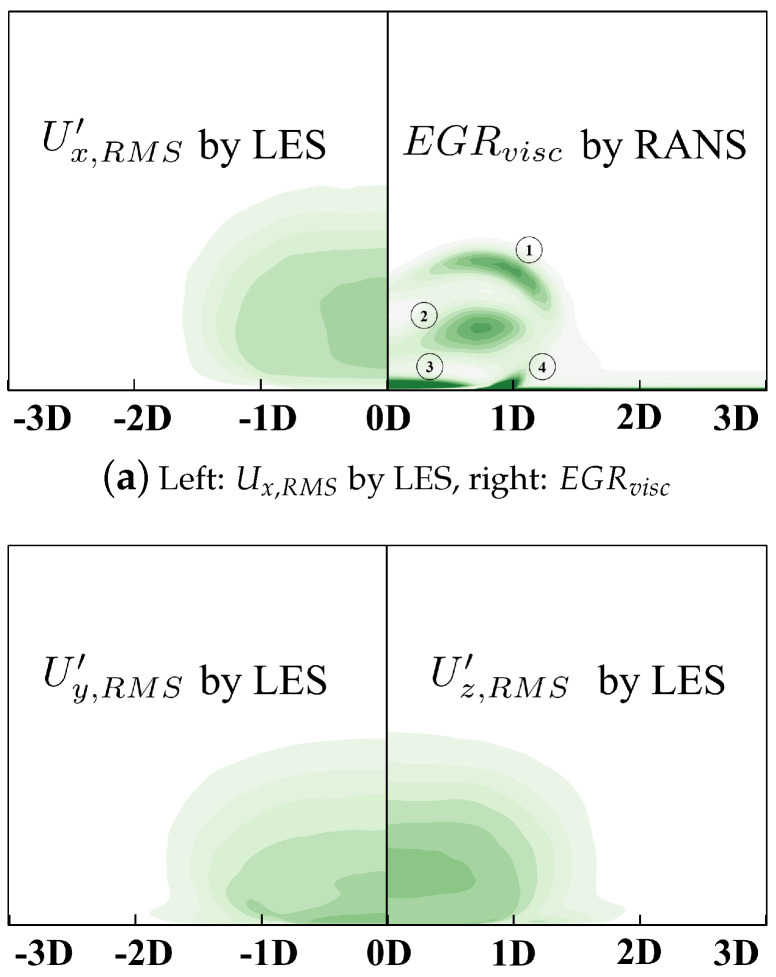
Demonstration of RMS velocity components calculated by LES at *z* = 0*D*, *M* = 3.0.

$T_{RMS}^{\prime }$ denotes the local fluctuation of the temperature which, to a certain extent, represents local intensity of the irreversible thermal mixture. The high-EGR region locates at the boundary between the coolant and mainflow in RANS, which can be correspondingly observed in the $T_{RMS}^{\prime }$ contour by LES. Similarly, the fluctuation of velocity components are demonstrated in Figure 18.

There are four high-EGR regions observed in Figure 18a. Region ① locates at the boundary between the coolant and main flow where shear action contributes to EGR intensity there. Region ② is in the core region of coolant which can be attributed to the high $U_{x,RMS}^{\prime }$ and $U_{z,RMS}^{\prime }$ there. EGR in Region ③ can be correspondingly ascribed to $U_{y,RMS}^{\prime }$. Region ④ lies near the no-slip wall, which can be due to the distortion of the boundary layer. We believe the results can reasonably stand on the conclusion that the RANS simulation is able to resolve the local high-EGR or low-EGR and reveal the underlying mechanism.

## 5. Effect of Passage Vortex on Film Cooling Performance

As has been revealed in the above analyses, vortical structures can significantly affect the coolant/mainstream mixture process and thus altering distributions of the cooling effectiveness. Ligrani et al. [24,25,26] ascribed this effect into the secondary-flow velocity vector field induced by the passage vortex. However, due to the limitation of the experimental resolution, there lack further analyses on the interaction of the second-order vortex interaction. In the current section, the interaction mechanism between the passage vortex and the kidney vortices is emphasized to account for the migration mechanism of coolant under the effect of vortex entrainment.

### 5.1. Effect of Hole Location

In this part, the effect of PV on the cooling performance is quantified by varying the hole-to-PV positions, and the underlying interaction mechanism is revealed by analyzing spatial distributions of the local entropy generation rate. The blowing ratio *M* is fixed at 1.0 in the current subsection and further discussions on *M* will be included in Section 5.2.

**Figure 19:**
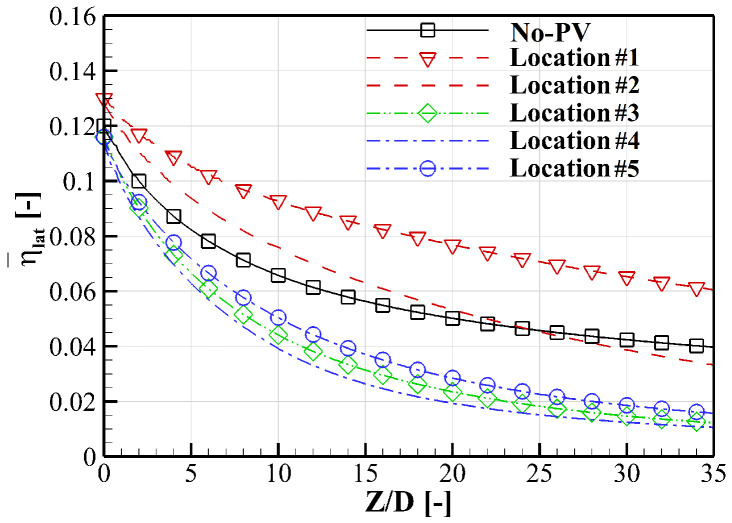
Laterally averaged cooling effectiveness distributions at *M* = 1.0.

**Figure 20:**
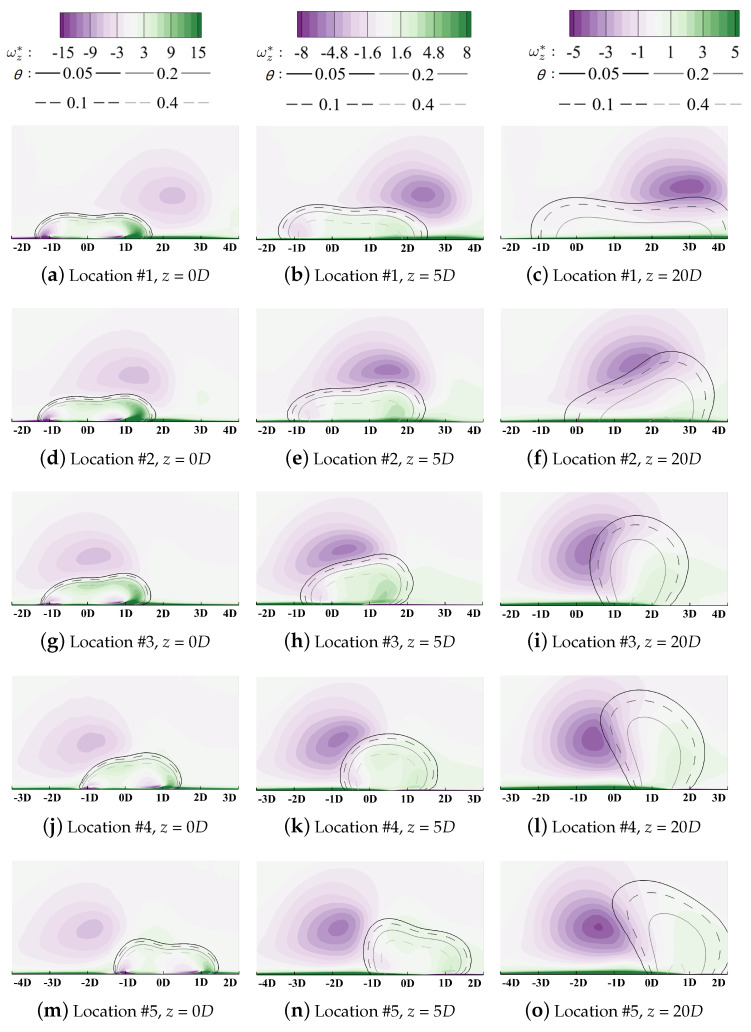
Contour of vorticity with isolines of non-dimensionalized temperatures at *M* = 1.0.

**Figure 21:**
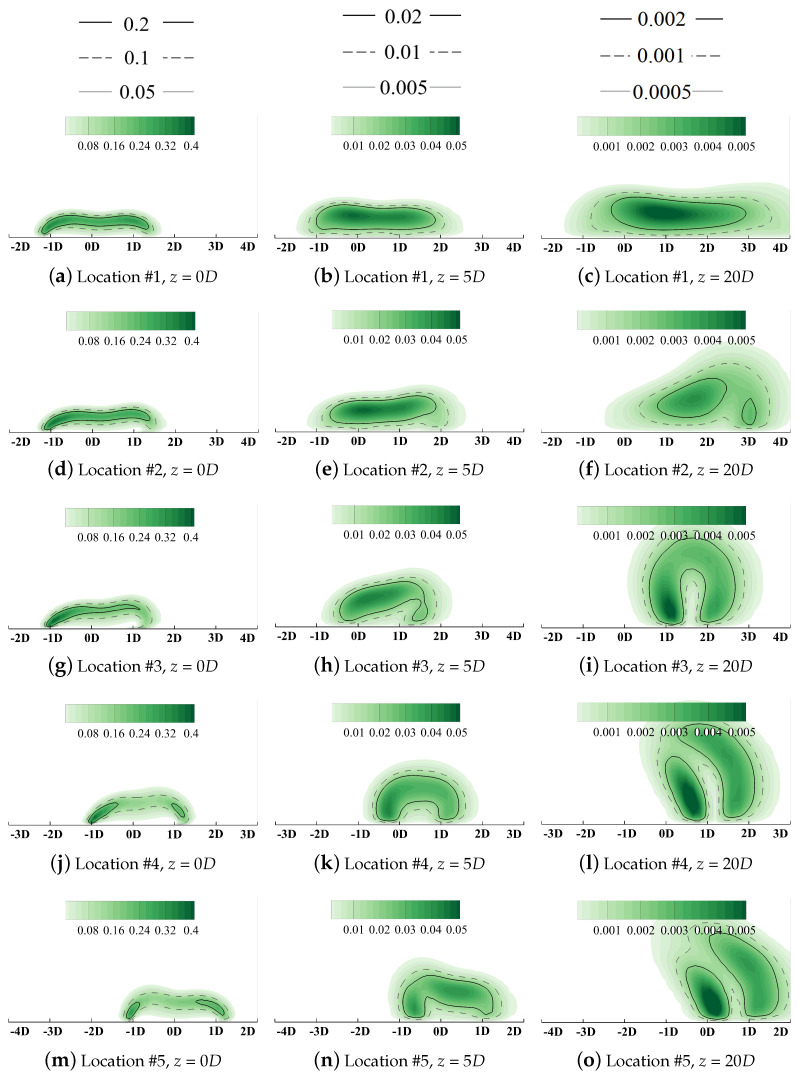
Contour of thermal entropy generation rate, $EGR_{ther}$, at *M* = 1.0.

Figure 19 plotted the laterally averaged cooling effectiveness (over the shaded region in Figure 3) along the streamwise direction for quantitative comparison. Red lines denote the film cooling in the downwash region and the blue lines are those in the upwash region. The reference case labeled “No-PV” represents the single hole film cooling on a flat plate, as given in Section 4.2. Generally, the existence of PV can both improve or deteriorate cooling performance determined by the relative position between PV and the cooling hole. For a more intuitive comprehension, spatial distributions of coolant are demonstrated using isolines in Figure 20 and the volumetric rate of thermal entropy generation is contoured in Figure 21.

Compared with the reference condition in Figure 15, the lateral film coverage is significantly enhanced when the hole centerline is located at Location #1 ($y_{r}=0D$ in these figures), as shown in Figure 20a–c. When coolant is ejected from the cooling hole, coolant covers the region from $y_{r}=-1.5D$ to 1.5D in Figure 20a. Then, due to the combined effects of the KV entrainment and the coolant lateral expansion, the left bound of coolant experiences slight migration from $y_{r}=-1.5D$ to −1D. For the right bound, it moves laterally towards $y_{r}=4D$ along the rotation direction of PV in Figure 20c. Cooling performance is improved significantly by enhancing coolant lateral spreading. Contours of the entropy generation rate are illustrated in Figure 21a–c. The negative leg KV${}_{n}$ does not directly interact with the large-scale PV spatially and the positive leg KV${}_{p}$ lies beneath PV. However, the coolant conserves its structure during the interaction process along the streamwise direction. The vortical interaction between PV and KV${}_{p}$, which have opposite rotation directions, does not strengthen the mixture process. Maximum heat transfer occurs in the region laterally from $y_{r}=-1D$ to 0D, instead of the direct-interaction region. Then, similar to that of the reference case in the flat plate, the KV structure dissipates along the streamwise direction and the maximum entropy generation occurs in the core of the coolant where the thermal diffusion is driven by the vertical temperature gradient dominates the heat transfer process. The coolant is fully attached to the wall surface and the lateral spreading is enhanced by PV, thus resulting in improved cooling performance.

For Location #2, the difference is smaller by comparing its laterally averaged cooling effectiveness with the reference no-PV condition. Coolant migrates laterally from $y_{r}=0D$ to 2D while the width of the film coverage remains almost unchanged in Figure 20d–f. During the interaction process between vortices with the same rotation direction, the larger-scale PV eats the weaker KV${}_{n}$, and the coolant is entrained inside the main flow. Then, two cores of the entropy generation can be observed in Figure 21f. The larger-size one represents the mixture region where the coolant is lifted off the surface. The other denotes the entrainment effect due to the combined KV${}_{p}$ and the positive-rotating-direction vortex induced by PV. It should be clarified that the absolute value of the entropy generation does not represent the degradation of the wall cooling effectiveness but the strength of the spatial irreversible heat transfer. When the coolant from Location #2 is lifted off the surface, the coolant accumulates in the upwash region, and the temperature gradient is smaller than that of Location #1. Despite its higher cooling capacity, the lifted coolant lacks the ability to lower the temperature of the wall surface, leading to deteriorated cooling effectiveness.

When the hole location moves along the lateral direction towards Location #3 and #4, this mixture effect becomes severer. As has been discussed, the interaction between vortices with the same rotation is the main cause that results in coolant being entrained into the main flow. The left bound of the coolant moves from $y_{r}=-1.5D$ towards 0.5D while the right bound experiences slight migration compared with the reference flat plate condition. Then, the lateral coverage by the coolant is shrunk significantly and the averaged cooling effectiveness bottoms at Location #4. By observing contours of the entropy generation rate in Figure 20g–l, the importance of the vortical interaction can be validated. It enhances the mixture process by entraining coolant inside the main flow and forms a loss core of the cooling capacity. Instead of attaching to the wall and preventing the component surface from directly exposing the high-temperature main gas, the coolant lowers the recovery temperature by lowering the near-wall main-flow temperature. This reduces the power capacity of the working medium. Then, for Location #5, due to the limited coolant/PV overlap region between the coolant and the main flow, the cooling effectiveness recovers slightly. Generally, when the cooling hole is located where the core region of PV overlaps with KV${}_{n}$, the coolant is fully lifted off the wall surface and the overall cooling performance is insensitive to changes in the hole location.

### 5.2. Effect of Blowing Ratio

**Figure 22:**
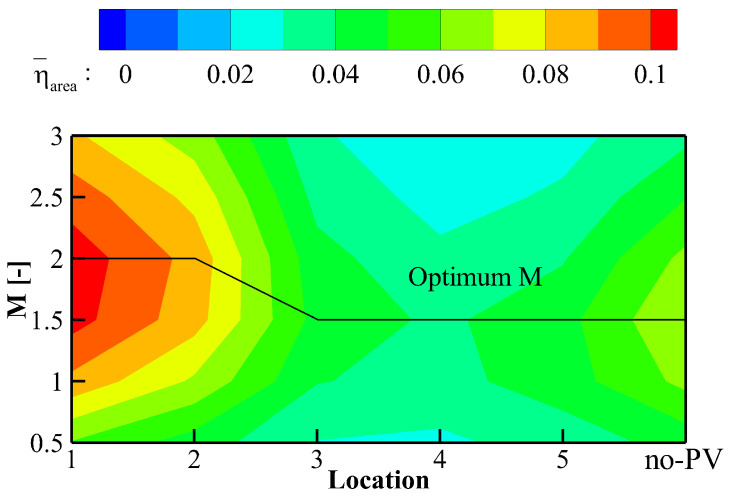
Area-averaged cooling effectiveness under varying blowing ratios and pitchwise locations.

Based on the above analyses, the effect of PV on the cooling performance can be mainly attributed to the enhancement or deterioration of the lateral spreading of coolant. Increasing the blowing ratio represents greater cooling capacity while it strengthens the momentum of the injection, leading to a higher tendency of coolant detachment and stronger intensity of KV. As a result of these competing effects, an optimum blowing ratio can be expected. The cooling effectiveness is averaged over the shaded region in Figure 3 and the results are contoured in Figure 22 using the location index as the horizontal axis and the blowing ratio as the vertical axis. The black solid line marks the optimum blowing ratio where maximum area-averaged effectiveness value can be obtained within the research range at a given hole location.

**Figure 23:**
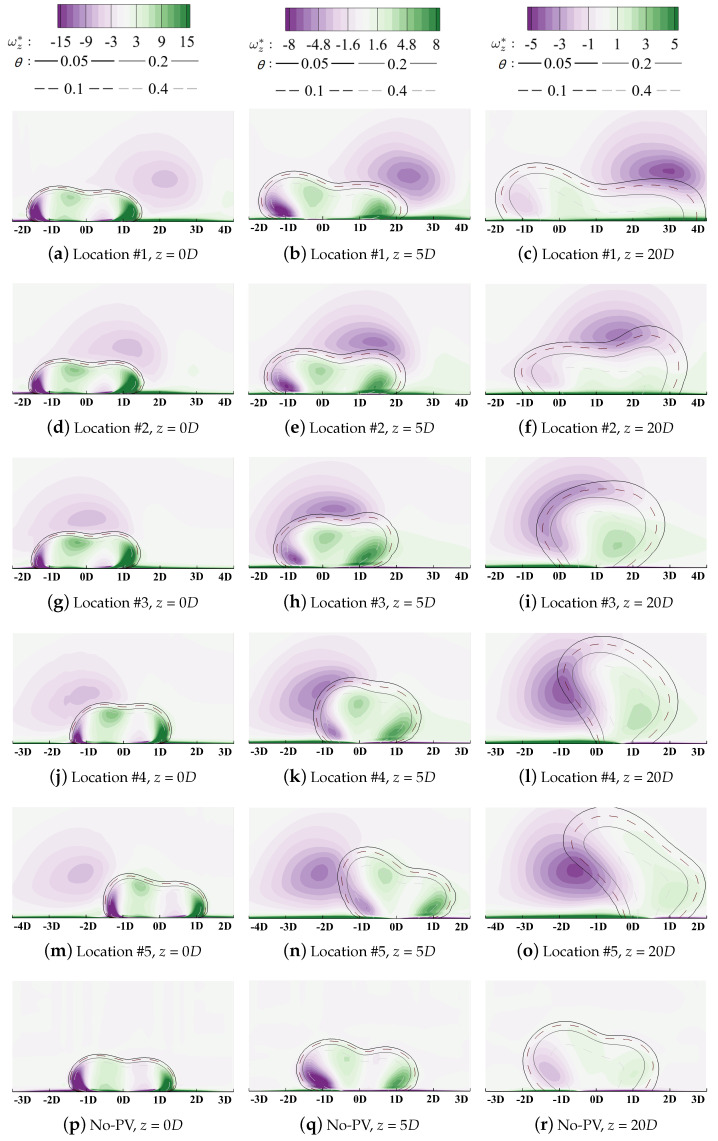
Contour of vorticity with isolines of non-dimensionalized temperatures at *M* = 2.0.

For the reference condition, the cooling performance tops at *M* = 1.5. Similar results can be obtained at Location #3 to #5 where a higher blowing ratio strengthens the injection momentum. However, higher overall cooling performance and higher optimum blowing ratio value are obtained when the cooling hole is located at Location #1 and #2. The vorticity contours are illustrated in Figure 23 to demonstrate the coolant/mainstream interaction at the high blowing ratio, *M* = 2.0 as an example, conditions. For the reference case in Figure 23p–r, it can be seen that the KV structure is strengthened. As a result, the coolant lateral spreading is suppressed compared with that at the low blowing ratio condition. A pair of induced counter-rotating kidney vortices is observed, which is labeled “CRKV” in the current paper. CRKV lies between two legs of KV with opposite rotating directions.

When the cooling hole locates in the downwash region at Location #1, the counter-rotating KV${}_{p}$ and PV squeezes each other and KV${}_{p}$ quickly dissipates during the interaction process in Figure 23c. Interaction between the large-scale PV and KV${}_{n}$ is cut off by the clockwise CRKV${}_{p}$. It can be seen that the left bound of the coolant remains almost unchanged by comparing Figure 23c with Figure 23r of the reference case. Then, the overall cooling performance is improved by laterally enhancing spreading coolant in the KV${}_{p}$-dominant region. When the coolant is ejected from Location #2, the effect of the PV/KV${}_{n}$ interaction can be observed with the left bound migrating laterally. The phenomenon can be observed that the development of the PV structure is suppressed by the high-momentum injection in Figure 23f but PV still dominates the flow field and enhances the lateral spreading of coolant. Similar results can be observed at Location #3 in Figure 23i where PV is deformed and merges with KV${}_{n}$. Another clockwise vortex structure forms on the right side. Due to the vortical entrainment, the lateral coverage of coolant shrinks, and the cooling performance deteriorates further. When the cooling hole is located in the upwash region at Location #4 and #5, the anti-clockwise PV structure is significantly enhanced by KV${}_{n}$. The averaged cooling effectiveness bottoms at Location #4, similar to that of the low blowing ratio condition.

Generally, the interaction mechanism between the cooling injection and the passage vortex revealed in the above discussions can well explain the enhancement or deterioration of cooling performance despite changes in the blowing ratio. However, the blowing ratio affects the absolute value of the overall cooling effectiveness and the optimum cooling performance is obtained at higher *M* when the cooling hole is located in the downwash region.

## 6. Conclusions

The main work of the current study is to reveal the interaction mechanism between the cooling injection and the passage vortex. In the current study, the model of a blunt body with a half-cylinder is introduced in a straight channel to generate the vortex structure. In this way, the effect of the passage vortex can be separated free from the flow acceleration and the crossflow. The reliability of the numerical results is validated using the experimental data with limiting streamlines for the aerodynamic prediction and the laterally averaged effectiveness for the cooling performance prediction. The entropy generation rate-based loss analysis method is introduced to quantify the intensity of the local irreversible loss. There are two sources, thermal irreversibility, and viscous irreversibility, that contribute to the local entropy generation. Based on the scale analysis on the magnitude, it is revealed that the thermal entropy generation dominates the interaction process between the cooling injection and the main flow.

The passage vortex has a significant effect on the film cooling performance. The film coverage can be improved or deteriorated determined by the relative location between the cooling hole and the passage vortex core. When the cooling hole is located at the downwash region, the lateral spreading of coolant is enhanced along the rotating direction of the passage vortex. However, the coolant ejected at the upwash region is quickly entrained into the passage vortex instead of attaching to the wall surface. The vortical interaction between the passage vortex and the kidney vortex pair is validated to have a significant effect on the mixture between the coolant and the main flow. Vortices with the same rotating direction, the passage vortex, and the negative leg of the kidney vortex pair, merge together, leading to enhanced irreversible heat transfer. The positive leg of the kidney vortex pair can survive with the passage vortex, and the coolant is able to conserve its structure. Changes in the blowing ratio have a significant effect on the cooling effectiveness, and on the enhancement or deterioration of the cooling performance compared with the flat plate film cooling.

## Figures and Tables

**Table 1:** Relevant parameters in the current study.

Parameter	Value	Parameter	Value
$D_{m}$	150 mm	*D*	7.75 mm
h/D	3.0	L/D	2.5
$\beta_{fwd}$	$7^{\circ }$	$\beta_{lat}$	$7^{\circ }$
α	$30^{\circ }$	*M*	0.5, 1.0, 1.5, 2.0, 2.5, 3.0
$U_{0}$	22 m/s	DR	1.5

## Nomenclature

Be	Bejan number
Cp	Specific heat
*D*	Diameter of the cooling hole
$D_{m}$	Diameter of the half-cylinder
DR	Density ratio, $\frac{\rho_{c}}{\rho_{0}}$
EGR	Non-dimensionalized entropy generation rate
*h*	Wall thickness
*I*	Momentum ratio
*k*	Thermal diffusion conductivity
*K*	Acceleration parameter
*L*	Hole length
*M*	Blowing ratio, $\frac{\rho_{c}\;\cdot \;U_{c}}{\rho_{0}\;\cdot \;U_{0}}$
Ma	Mach number
Pr	Prandtl number
*s* or *S*	Entropy
$\dot{S}$	Entropy generation rate
*T*	Temperature
*U*	Velocity
*V*	Volume
*x* or *X*	Spanwise direction
*y* or *Y*	Pitchwise direction
*z* or *Z*	Streamwise direction
*Greek*	
α	Injection angle
$\alpha_{t}$	Turbulent thermal diffusion coefficient
β	Expansion angle
η	Adiabatic cooling effectiveness, $\frac{T_{aw}-T_{0}}{T_{c}-T_{0}}$
γ	Deviation angle
λ	Thermal conductivity
ν	Viscosity
$\omega^{*}$	Non-dimensionalized vorticity, $\frac{\omega }{U_{0}/D_{m}}$
$\Phi_{\Theta }$	Thermal irreversible entropy generation
ρ	Density
$\tau_{c}$	Characteristic time scale
$\tau_{i,j}$	Viscous stress
θ	Non-dimensionalized temperature, $\frac{T-T_{0}}{T_{c}-T_{0}}$
ε	Tensor of the deformation rate
*Subscript*	
aw	Adiabatic wall
*c*	Coolant
fwd	Forward direction
lat	Lateral/pitchwise direction
0	Main-flow
*t*	Turbulent
ther	Thermal
visc	Viscous
*Abbreviation*	
BL	Boundary layer
PV	Passage vortex
